# Macular perfusion analysis using optical coherence tomography angiography post rhegmatogenous retinal detachment repair: a systematic review and meta-analysis

**DOI:** 10.1007/s10792-026-04210-8

**Published:** 2026-08-20

**Authors:** Abdullah Ahmed, Fiza Shaheen, Hashem Abu Serhan, Zain Ali Nadeem, Mahrukh Chaudhry, Eeman Ahmad, Hasan Khan, Shahzaib Ahmed, Umar Akram, Muhammad Talha, Obaid Ur Rehman, Ayman G. Elnahry, Muhammad Amer Awan

**Affiliations:** 1 Department of Ophthalmology and Visual Sciences, University of Illinois Chicago, Chicago, IL USA; 2 Department of Medicine, Allama Iqbal Medical College, Lahore, Pakistan; 3 Department of Ophthalmology, Shifa International Hospital, Islamabad, Pakistan; 4 Department of Ophthalmology, Hamad Medical Corporation, Al-Rayyan St., 3050 Doha, Qatar; 5 Department of Medicine, Fatima Memorial Hospital College of Medicine and Dentistry, Lahore, Pakistan; 6 Shaikh Khalifa Bin Zayed Al-Nahyan Medical and Dental College, Lahore, Pakistan; 7 Department of Medicine, Services Institute of Medical Sciences, Lahore, Pakistan; 8 Department of Ophthalmology and Visual Sciences, School of Medicine and Public Health, University of Wisconsin, Madison, WI USA; 9 Department of Ophthalmology, Faculty of Medicine, Cairo University, Cairo, Egypt; 10 BayCare Clinic Eye Specialists, Aurora BayCare Medical Center, Green Bay, WI USA

**Keywords:** Optical coherence tomography angiography, Retinal detachment, Vitrectomy, Macula, Microvasculature

## Abstract

**Background:**

Evaluating rhegmatogenous retinal detachment (RRD) repair traditionally focuses on anatomical reattachment and visual acuity. Understanding macular perfusion changes post-repair offers a more comprehensive assessment. Optical coherence tomography angiography (OCTA) non-invasively visualizes retinal microvasculature, enabling detailed perfusion analysis.

**Methods:**

Our protocol was registered (PROSPERO CRD42024533072). We searched PubMed, Scopus, ScienceDirect, and Cochrane databases (from inception to November 2024) for studies comparing OCTA parameters between post-repair RRD eyes and controls. Analyzed parameters included foveal avascular zone (FAZ) area, foveal (FVD) and parafoveal vascular density (PFVD), choriocapillaris (CC) perfusion, and best-corrected visual acuity (BCVA). Data were analyzed for macula-ON and macula-OFF groups using a random-effects model.

**Results:**

Twenty studies comprising 686 RRD eyes (231 macula-ON, 455 macula-OFF) and 686 control eyes were analyzed. In macula-OFF cases, FAZ area remained significantly enlarged post-repair in both superficial capillary plexus (SCP) (0.06 mm^2^, 95% CI: 0.02–0.09) and deep capillary plexus (DCP) (0.12 mm^2^, 95% CI: 0.04–0.19). FVD showed no significant differences in either group. PFVD demonstrated a significant reduction in DCP for both macula-ON (− 1.38%, 95% CI: − 2.72 to − 0.05) and macula-OFF (− 2.83%, 95% CI: − 5.04 to − 0.63) groups. CC perfusion density showed a significant reduction post-repair (− 2.39%, 95% CI: − 4.56 to − 0.22). Postoperative BCVA improved significantly in macula-OFF cases (0.79 logMAR, 95% CI: 0.37–1.21) but showed no significant change in macula-ON cases.

**Conclusions:**

Despite successful anatomical repair of RRD, persistent alterations in macular perfusion parameters were observed, particularly in the macula-OFF group. The enlarged FAZ area and reduced PFVD and CC perfusion density suggest lasting changes in the retinal microvasculature post-repair. However, significant improvement in BCVA, especially in macula-OFF cases, indicates that functional recovery may occur despite these vascular changes. These findings highlight the need for comprehensive assessment beyond isolated perfusion metrics when evaluating surgical success in RRD repair.

**Supplementary Information:**

The online version contains supplementary material available at 10.1007/s10792-026-04210-8.

## Introduction

Rhegmatogenous retinal detachment (RRD) is a significant ophthalmic emergency characterized by the separation of the neurosensory retina from the underlying retinal pigment epithelium (RPE) [1]. This detachment occurs when liquefied vitreous humor infiltrates through a retinal tear or hole, leading to potential vision loss if not promptly addressed [2]. The incidence of RRD varies geographically, with studies reporting rates between 6.3 and 17.9 per 100,000 individuals annually [3]. The pathophysiology of RRD involves vitreous degeneration and posterior vitreous detachment, which exert traction on the retina, resulting in tears that allow subretinal fluid accumulation and subsequent retinal separation [4].

The retinal microvasculature is crucial for the structural integrity and functional recovery in RRD-affected eyes. RRD can disrupt retinal microvasculature, causing macular ischemia that impacts visual outcomes even after successful retinal reattachment. Optical coherence tomography angiography (OCTA), a non-invasive imaging technique, enables detailed visualization of retinal capillary layers—superficial capillary plexus (SCP), deep capillary plexus (DCP), and choriocapillaris plexus (CCP) without contrast agents [5, 6]. OCTA offers advantages over fluorescein angiography, providing depth-resolved images without dye-related risks [7]. Even when conventional OCT shows repaired retinal microstructure without apparent abnormalities, patients may experience functional declines like color vision defects and persistent metamorphopsia [8]. OCTA enables visualization of retinochoroidal microvasculature and quantitative analysis of macular vessel density (VD) and foveal avascular zone (FAZ) area, helping examine mechanisms behind poor RRD prognosis.

Recent studies utilizing OCTA have investigated macular perfusion changes following RRD repair. For instance, Wang et al. [5] conducted a study involving 14 eyes with macula-off RRD that underwent PPV. They observed a significant increase in flow densities of the SCP, DCP, and choriocapillaris over a 12-week postoperative period, indicating gradual recovery of macular perfusion. Furthermore, they reported a positive correlation between final best-corrected visual acuity (BCVA) and choriocapillaris flow density, suggesting that improved macular perfusion is associated with better visual outcomes. Similarly, another study [9] examined the relationship between macular perfusion and long-term visual outcomes in patients who had undergone successful surgical repair of macula-off RRD. Their findings indicated that reduced vessel density in the SCP and DCP, as well as an enlarged FAZ area, were associated with poorer visual recovery. Despite these insights, the literature presents variability in findings regarding the extent and timeline of macular perfusion recovery post-RRD repair. Factors such as the type of surgical intervention, duration of retinal detachment, and presence of preoperative macular involvement may contribute to these discrepancies. For example, Tsen et al. [10] reported that eyes with preoperative intraretinal separation exhibited a larger FAZ and lower DCP vessel density postoperatively, highlighting the impact of preoperative retinal status on microvascular outcomes.

The primary objective of this meta-analysis is to systematically review and consolidate studies utilizing OCTA to assess macular perfusion changes following RRD repair. Through this comprehensive analysis, we aim to provide a clearer understanding of the vascular dynamics involved in RRD repair and their implications for visual function.

## Materials and methods

### Study protocol

We conducted this meta-analysis following the guidelines outlined by the Preferred Reporting Items for Systematic Reviews and Meta-Analyses (PRISMA 2020) [11]. The review is registered with the International Prospective Register of Systematic Reviews (CRD42024533072). Our review adhered to the tenets of the Declaration of Helsinki.

### Eligibility criteria

We included (1) studies involving participants with RRD in one eye, (2) studies reporting successful reattachment of the detached retina after a single surgical procedure, (3) studies that classify data based on whether the macula was detached (macula-OFF) or attached (macula-ON) at the time of the procedure, (4) studies that include comparisons with healthy fellow eyes or control eyes, (5) studies utilizing OCTA to assess retinal microvasculature, and (6) studies published in the English language. On the other hand, we excluded (1) studies involving cases where eyes required multiple surgeries for RRD repair, (2) studies lacking control groups or comparisons with fellow eyes, (3) studies including eyes with post-operative complications such as macular edema, macular cysts, or sub macular fluid, (4) studies involving participants with pre-existing macular impairments like diabetic retinopathy, choroidal detachment, or fibrous bands, and (5) studies conducted on animal models.

### Literature search and study selection

We conducted a thorough electronic bibliographic search to identify articles published from the inception to November 2024. We searched electronic databases/registers including PubMed, Scopus, ScienceDirect, and Cochrane for possible original articles using keywords and Medical Subject Headings (MeSH) terms such as “Optical Coherence Tomography Angiography,” and “Rhegmatogenous Retinal Detachment,”. A detailed search strategy is provided in Table S1. We examined the references of retrieved full-text articles to identify potentially relevant articles that may not have been detected through the electronic search.

Following the initial search, we removed duplicate articles using EndNote Reference Library Software. Two independent reviewers [AA, FS] screened titles and abstracts identified by the search strategy to assess eligibility. If the title and abstract did not provide sufficient information, the full text was retrieved and evaluated. Any discrepancies in study eligibility were resolved through discussion. The same two reviewers [AA, FS] conducted a full-text screening of all selected articles, including only those that met the eligibility criteria.

### Data extraction

Two independent reviewers [AA, MC] performed the data extraction using an Excel® spreadsheet. A third reviewer [MAA] conducted a comparative analysis and refinement of the raw data to ensure extraction accuracy. The extracted data encompassed several key parameters including the primary author’s name, publication year, country of origin, population characteristics, sample size, study design, and baseline characteristics. Main outcome measures from the relevant studies included FAZ area, vascular density in the foveal area (FVD), vascular density in the parafoveal area (PFVD), perfusion density in choriocapillaris (CC), and best-corrected visual acuity (BCVA). As per the included studies, the foveal area was described as a central circular region ranging from 1 to 1.5 mm in diameter, while the parafoveal area was defined as a circular region between 1 and 2.5–3 mm in diameter. We examined the FVD and PFVD. Data on BCVA were extracted from studies reporting BCVA measurements post-conversion from Snellen charts to the logarithm of the minimum angle of resolution (logMAR) for statistical analysis. Postoperative BCVA was compared with preoperative BCVA to determine the efficacy of surgical intervention, facilitating a direct comparison of visual acuity pre- and post-operation. Moreover, the size of OCTA scans exhibited considerable diversity across studies, although 3×3 mm or 6×6 mm configurations were frequently used.

### Quality and risk of bias assessment

We assessed the quality of selected studies using the Newcastle–Ottawa Scale (NOS) [12] because all the studies were non-randomized. This scale defined scores based on domains under three categories: Selection (0–4 stars), Comparability (0–2 stars), Outcomes (0–3 stars). We categorized studies based on their quality scores, with those scoring less than 6 deemed as low-quality, scores between 6 and 7 classified as intermediate-quality, and scores ranging from 8 to 9 designated as high-quality. The publication bias was assessed by Egger’s test and constructing the Doi plots with Luis Furuya-Kanomori (LFK) index, to detect and quantify asymmetry of study effects [13].

### Statistical analysis

We used the packages “meta” and “metasens” for all statistical analyses in R version 4.5.0. We calculated the mean differences (MD) for continuous outcomes using an inverse variance random-effects model, with the restricted maximum likelihood estimator for tau^2^ [14]. We created forest plots to assess the pooled results. We assessed heterogeneity among the studies by examining Higgins’ I^2^, considering a value under 50% as acceptable. To address heterogeneity, we conducted a subgroup analysis and a leave-one-out sensitivity (L1O) analysis omitting one study at a time (Table S2). We performed a chi-square test to assess the differences between the subgroups. It should be noted that BCVA analysis represents a fundamentally different analytical framework from the OCTA parameters: whereas OCTA outcomes (FAZ area, FVD, PFVD, CC perfusion) reflect between-group comparisons of postoperative RRD eyes versus control eyes, BCVA was analyzed as a within-subject pre-to-postoperative comparison using mean change scores (postoperative minus preoperative BCVA) extracted directly from each included study. The pooled mean difference for BCVA therefore represents the average within-eye change following surgery, not a comparison against controls.

## Results

### Baseline characteristics

**Fig. 1:**
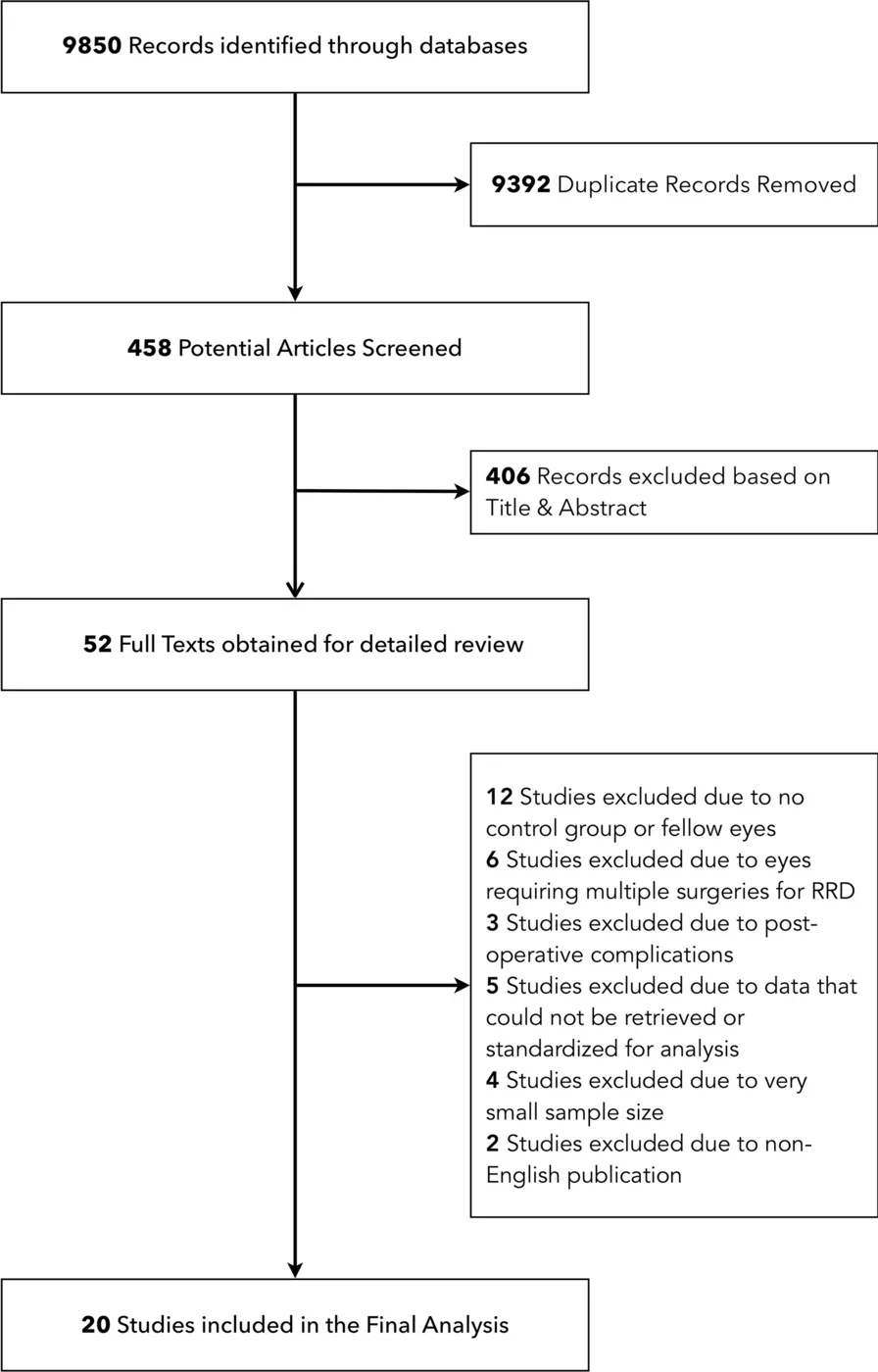
Preferred reporting items for systematic reviews and meta-analyses (PRISMA) flowchart

The PRISMA flowchart that summarizes the study selection procedure is depicted in Fig. 1. We analyzed a total of 20 studies [5, 15–33], comprising 686 RRD eyes alongside 686 control eyes. Among the RRD eyes, 231 were macula-ON, while 455 were macula-OFF. Notably, four studies [25, 28, 29, 32] exclusively enrolled participants with macula-ON RRD, while seven studies [5, 16, 20–22, 24, 31] focused on macula-OFF cases, and nine studies [15, 17–19, 23, 26, 27, 30, 33] reported a mix of both macula-ON and macula-OFF participants.

In a study conducted by Cetinkaya et al. [23], only 5 out of 32 participants demonstrated macula-ON status, prompting the overall data to be classified as macula-OFF, in line with the study’s findings. Similarly, in the investigation by D’Aloisio et al. [25], a subset of RRD patients, in which a portion of macula was detached before surgery who underwent surgical treatment within 24 h of onset. As a result, this subgroup was categorized as macula-ON RRD for our analysis. PPV was the predominant standard of practice for treating retinal detachment across most studies. However, treatment modalities such as scleral buckling and pneumatic retinopexy were also noted in some of the studies, such as those conducted by Zabel et al. [29] and Kaderli et al. [26], respectively. The timeframe between surgical intervention and OCTA imaging showed significant variability across studies, ranging from a minimum of 3 weeks to a maximum of 36 months. Table 1 summarizes the baseline parameters of all selected studies.

**Table 1:** Baseline characteristics of all included studies

Study Name	Country	Study Design	RRD Eyes	Control Eyes	Age (Mean)	Gender (M/F)	Macular Status On	Macular Status Off	PPV	SB	PR	OCTA Time	OCTA Device	Scan Protocol
Diaz-Aljaro 2024 [31]	Spain	Prospective	44	44	68.9±11.8	33/11	0	44	44	–	–	1, 3 and 6 mo	DRI OCT Triton (Topcon Corporation, Tokyo, Japan)	3×3 mm
Quarta 2024 [32]	Italy	Prospective	21	21	60.09±10.22	NR	21	0	–	–	–	NR	Plex Elite 9000 (Carl Zeiss Meditech, Dublin, CA)	3×3 mm
Shaheen 2024 [33]	Pakistan	Prospective	8	8	53.38±13.92	6/2	2	6	8	–	–	3–12 w	DRI OCT Triton (Topcon, Tokyo, Japan)	4.5×4 5 mm
Zabel 2023 [29]	Poland	Retrospective	20	20	58.1±11.46	9/11	20	0	–	20	–	9.15±2.79 mo	Avanti RTVue XR (Optovue, Inc., Fremont, CA)	6×6 mm
Machairoudia 2023 [30]	Greece	Prospective	48	48	63.8±8.7	29/19	15	33	48	–	–	6 mo	Huvitz HOCT-1F (Huvitz, Korea)	4.5 mm, 384×384 A-scans
Chatziralli 2022 [22]	Greece	Prospective	89	89	67.9±5.7	53/36	0	89	89	–	–	5 w	AngioVue (Optovue, Fremont, CA)	8×8 mm
D’Aloisio 2022 [25]	Italy	Prospective	14	14	52.6±15.2	8/6	14	0	14	–	–	3 mo	Canon OCT HS100 (Canon Inc., Tokyo, Japan)	3×3 mm
Çetinkaya 2021 [23]	Turkey	Prospective	37	37	57.8±10.2	29/8	5	32	23	–	14	3 mo	RTVue XR100-2 (Optovue, Fremont, CA)	6×6 mm
Kaderli 2021 [26]	Turkey	Prospective	30	30	61.1±7.7	4/26	18	12	–	–	30	3 mo	RTVue (Optovue, Fremont, CA)	6×6 mm
Liu 2021 [28]	China	Retrospective	16	16	55.5±9.68	4/12	16	0	16	–	–	36.3±3.7 mo	RTVue-XR Avanti (Optovue Inc., Fremont, CA)	3×3 mm and 6×6 mm
Lee and Park 2021 [27]	Korea	Retrospective	48	48	57.6±13.1	27/21	23	25	48	–	–	3 mo	DRI OCT Triton (Topcon, Tokyo, Japan)	4.5×4 5 mm
Christou 2021 [24]	Greece	Retrospective	23	23	62.7±8.18	18/5	0	23	23	–	–	12 w	AngioPlex (ZEISS, Jena, Germany)	6×6 mm
McKay 2020 [21]	United States	Retrospective	17	17	57±11	11/6	0	17	17	–	–	4±4 mo	Avanti SD-OCT (Optovue, Inc., Fremont, CA)	3×3 mm
Barca 2020 [17]	Italy	Prospective	33	33	60.26±11.52	20/13	15	18	18	15	–	6 mo	RTVue XR Avanti (Optovue, Fremont, CA)	3×3 mm
Bonfiglio 2020 [18]	Italy	Retrospective	93	93	62±8	52/41	56	37	93	–	–	17.2±5.6 mo	XR Avanti AngioVue (Optovue)	6×6 mm
Ng 2020 [20]	Netherlands	Prospective	47	47	60±9	36/11	0	47	43	4	–	12 mo	Spectralis OCT2 (Heidelberg Engineering, Heidelberg, Germany)	3×3 mm
Hong 2020 [19]	Korea	Retrospective	31	31	53.3±14.4	23/8	11	20	31	–	–	6 mo	DRI OCT Triton (Topcon Corporation, Tokyo, Japan)	4.5×4 5 mm
Agarwal 2019 [16]	India	Prospective	19	19	40.21±16.81	15/4	0	19	11	8	–	3 mo	RTVue XR 100 Avanti (Optovue Inc., Fremont, CA)	3×3 mm
Wang 2019 [5]	China	Retrospective	14	14	56 (42–73)	8/6	0	14	14	–	–	12 w	RTVue XR Avanti (Optovue Inc., Fremont, CA)	3×3 mm
Woo 2018 [15]	Korea	Retrospective	34	34	55.4±11.1	20/14	15	19	34	–	–	<2 mo	DRI OCT-1 Atlantis (Topcon Corporation, Tokyo, Japan)	3×3 mm

**RRD**= Rhegmatogenous Retinal Detachment, **PPV**= Pars Plana Vitrectomy, **SB**= Scleral Buckling, **PR**= Pneumatic Retinopexy, **NR**= Not Reported, **mo**= months, **w**= weeks

### Foveal avascular zone (FAZ) area

**Fig. 2:**
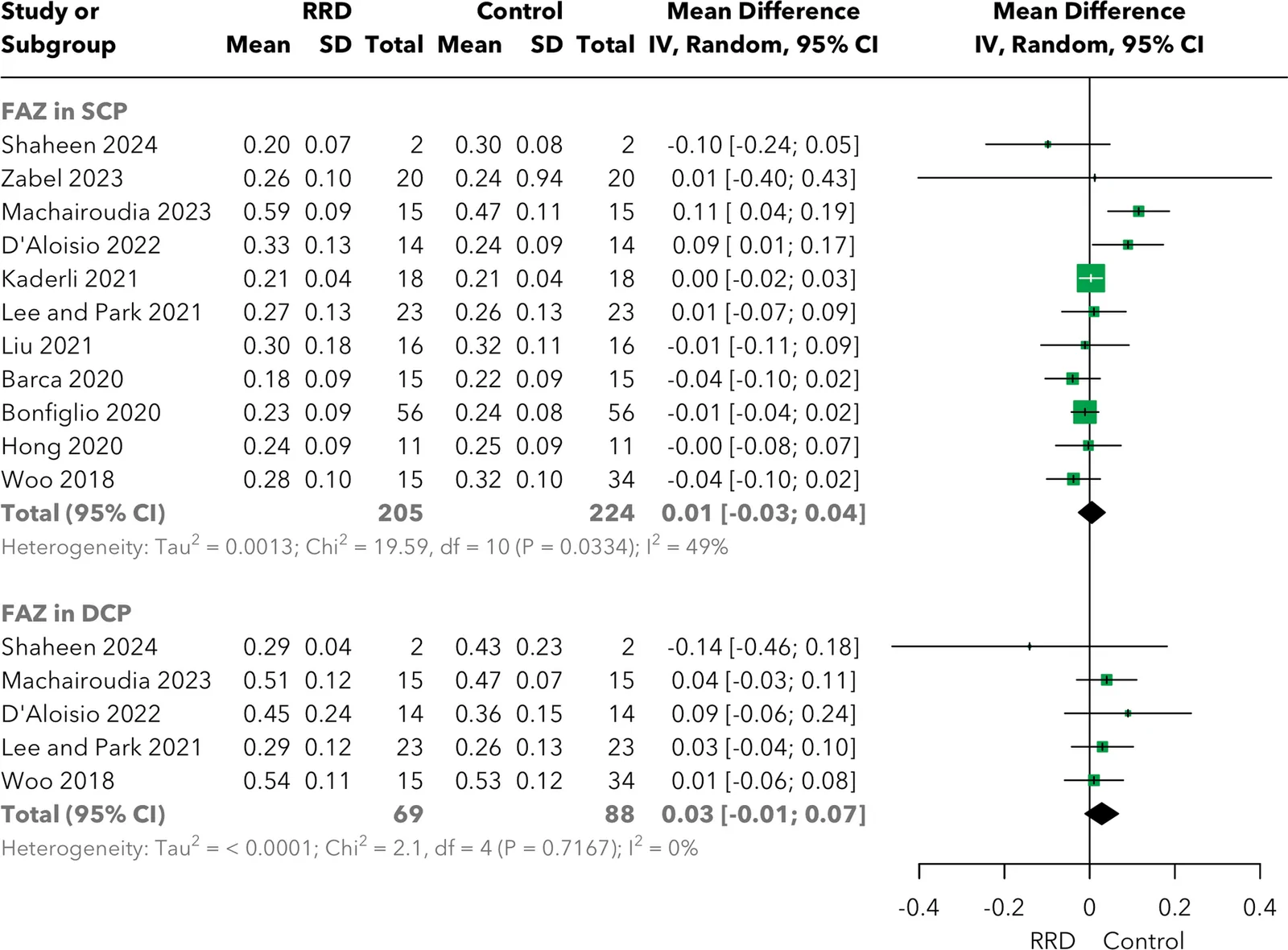
Foveal avascular zone (FAZ) area in macula-ON group

Analysis of the FAZ area was conducted in both macula-ON and macula-OFF groups across SCP and DCP. In the macula-ON cohort (n = 274 RRD eyes), as illustrated in Fig. 2, comparison with control eyes revealed no statistically significant differences in FAZ area for either the SCP (MD: 0.01 mm^2^, 95% CI: − 0.03 to 0.04) or DCP (MD: 0.03 mm^2^, 95% CI: − 0.01–0.07). Mild heterogeneity was observed in SCP measurements (I^2^ = 49%), while DCP measurements showed no heterogeneity (I^2^ = 0%).

**Fig. 3:**
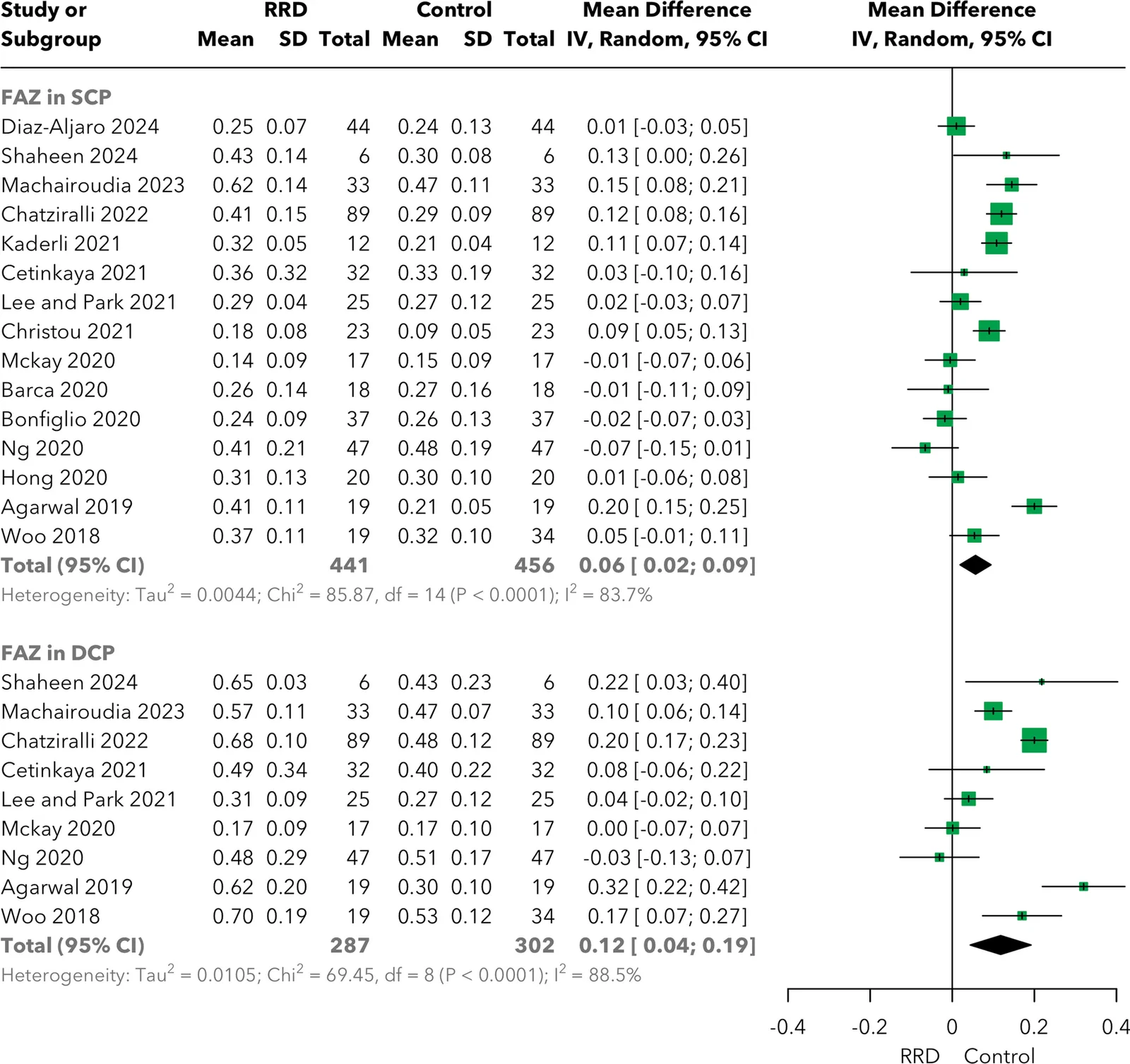
Foveal avascular zone (FAZ) area in macula-OFF group

The macula-OFF group analysis (n = 441 eyes for SCP; n = 287 eyes for DCP), depicted in Fig. 3, demonstrated persistently enlarged FAZ areas post-RRD repair in both the SCP (MD: 0.06 mm^2^, 95% CI: 0.02–0.09) and DCP (MD: 0.12 mm^2^, 95% CI: 0.04–0.19). Substantial heterogeneity was noted in both plexuses (I^2^ = 83.7% and 88.5%, respectively).

### Foveal vascular density (FVD)

**Fig. 4:**
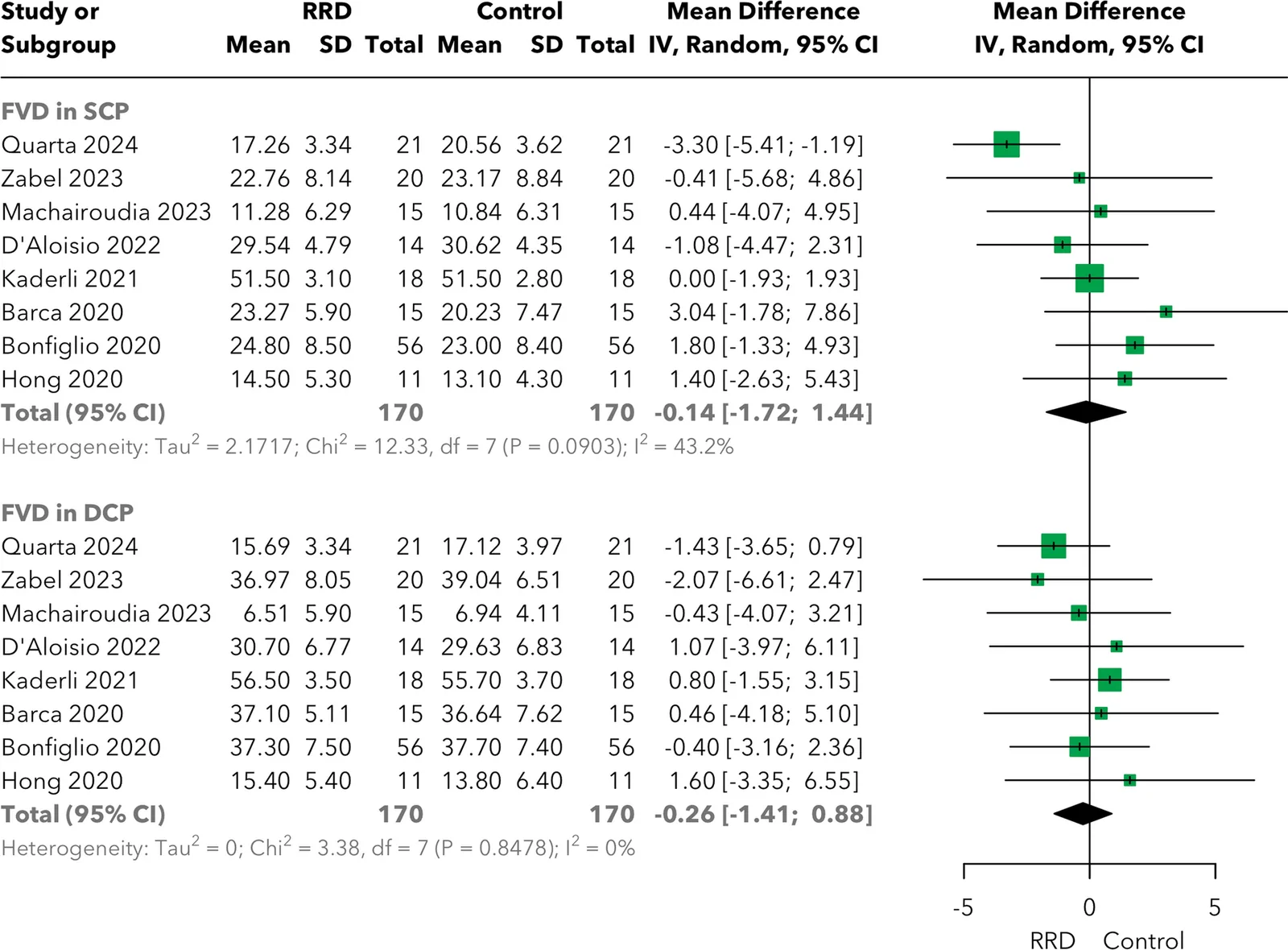
Foveal vessel density (FVD) in macula-ON group

FVD analysis in the macula-ON group (n = 340 eyes), as shown in Fig. 4, revealed no significant differences compared to controls in either the SCP (MD: − 0.14%, 95% CI: − 1.72–1.44) or DCP (MD: − 0.26%, 95% CI: − 1.41–0.88), with mild to no heterogeneity (I^2^=43.2% and 0%, respectively).

**Fig. 5:**
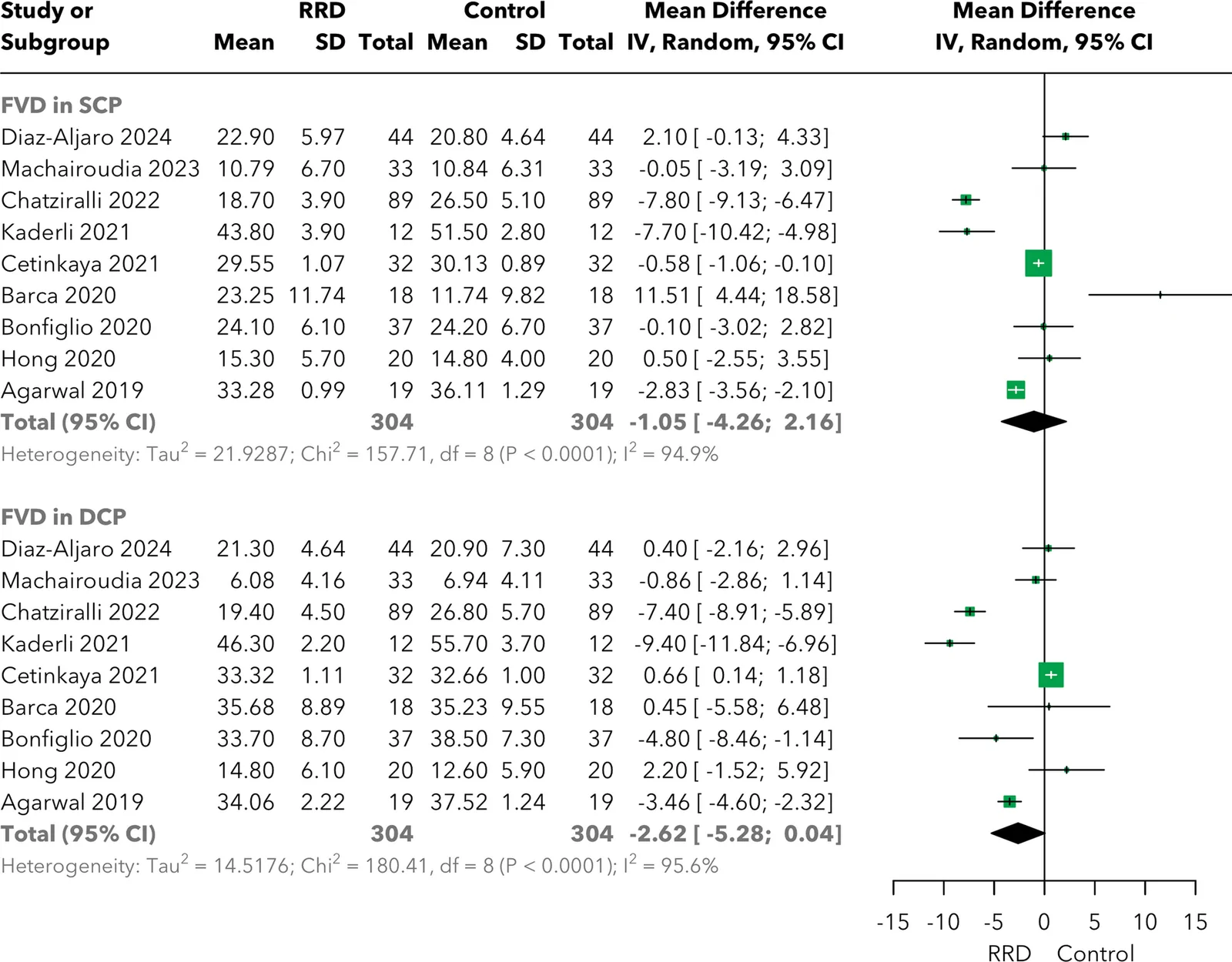
Foveal vessel density (FVD) in macula-OFF group

In the macula-OFF cohort (n = 608 eyes), Fig. 5 demonstrates that FVD measurements revealed no significant differences in either plexus (SCP: MD: − 1.05%, 95% CI: − 4.26–2.16; DCP: MD: − 2.62%, 95% CI: − 5.28–0.04). However, substantial heterogeneity was observed (SCP: I^2^=94.9%; DCP: I^2^=95.6%).

### Parafoveal vascular density (PFVD)

**Fig. 6:**
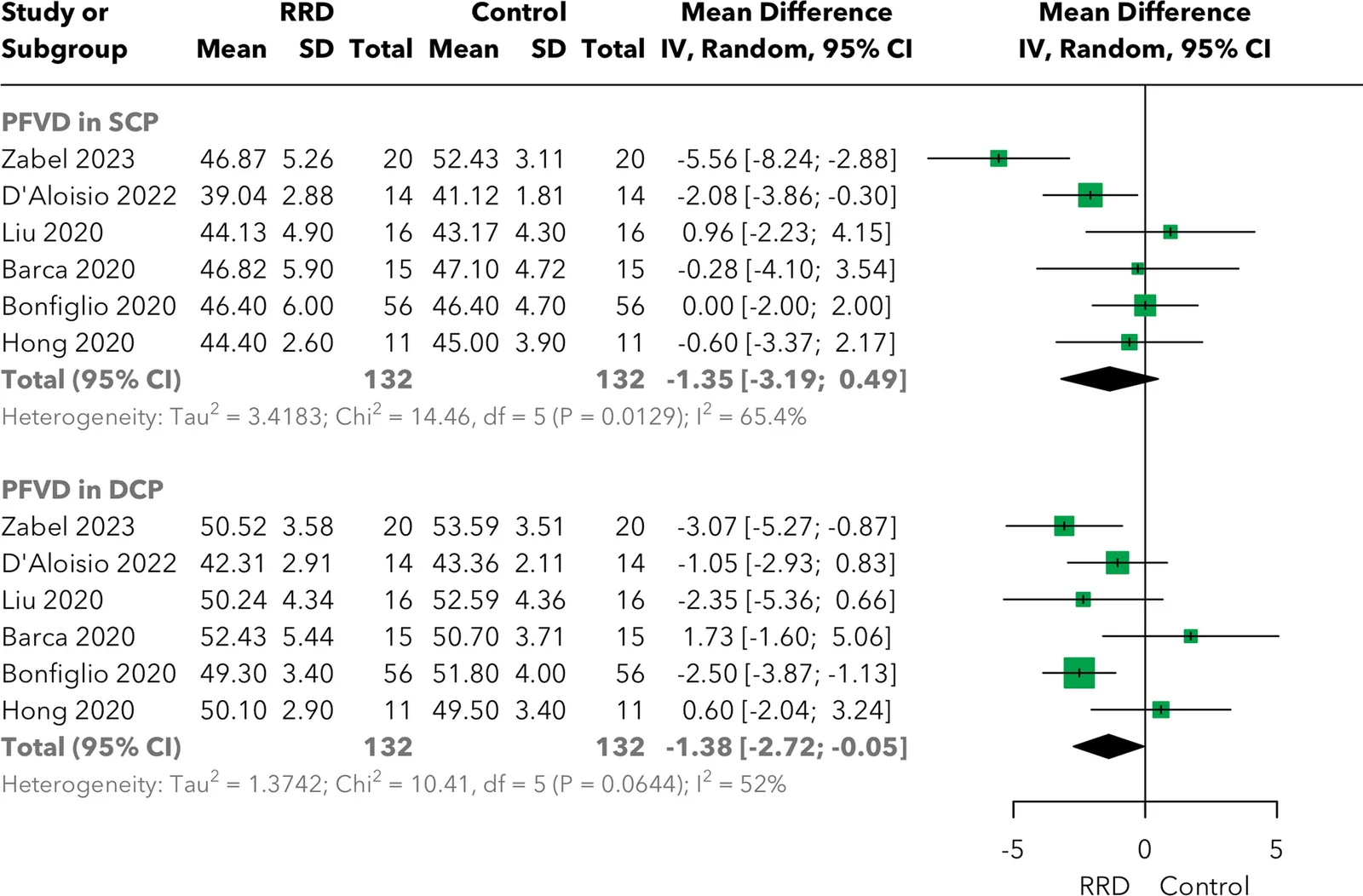
Parafoveal vessel density (PFVD) in macula-ON group

Analysis of PFVD in the macula-ON group (n = 264 eyes) was evaluated, and as shown in Fig. 6, there was no significant difference in SCP (MD: − 1.35%, 95% CI: − 3.19–0.49) but a significant reduction in DCP (MD: − 1.38%, 95% CI: − 2.72 to − 0.05). Both measurements demonstrated considerable heterogeneity (I^2^=65.4% and 52%, respectively).

**Fig. 7:**
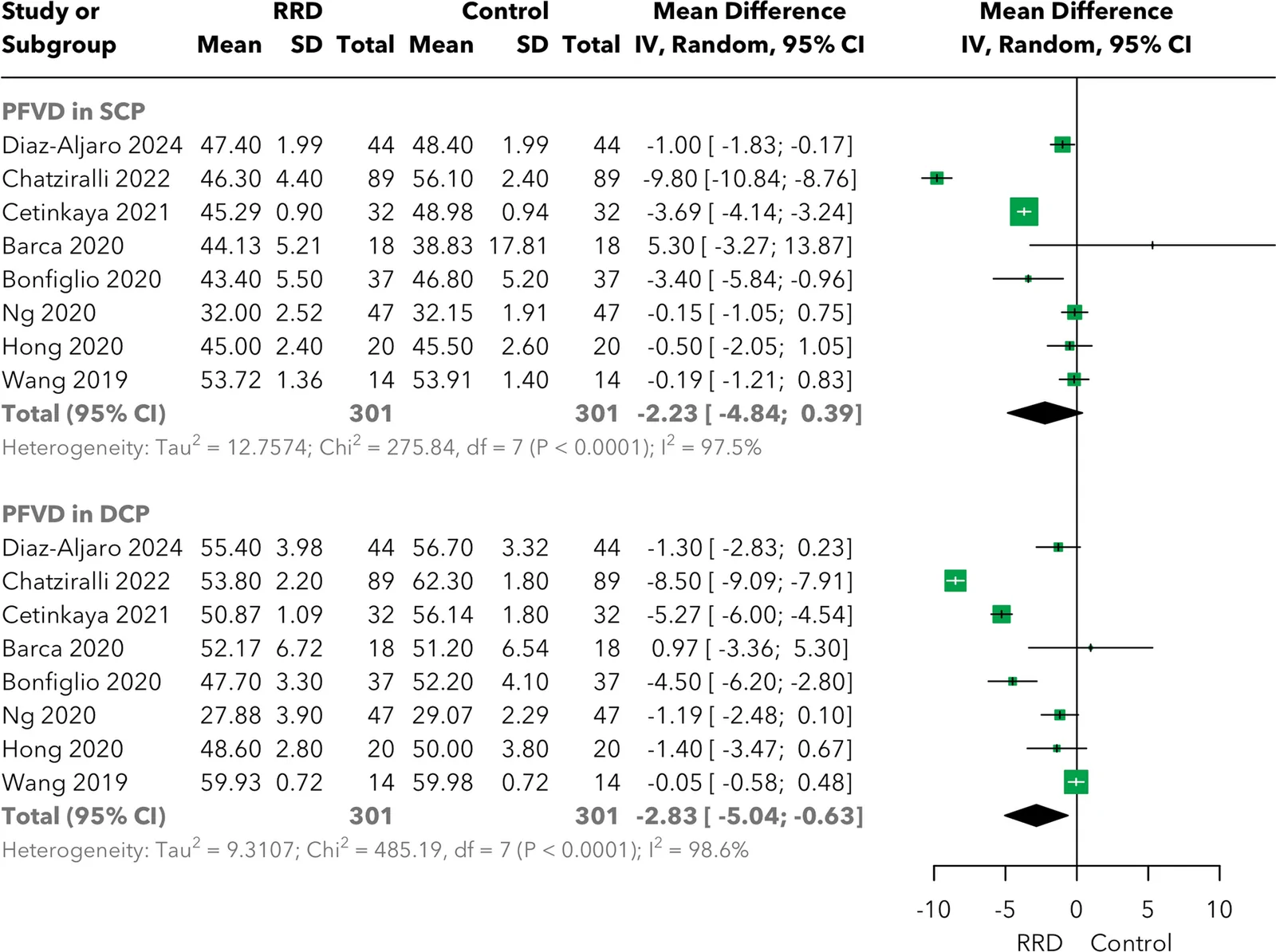
Parafoveal vessel density (PFVD) in macula-OFF group

The macula-OFF group (n = 602 eyes), illustrated in Fig. 7, exhibited non-significant PFVD reduction in SCP (MD: − 2.23%, 95% CI: − 4.84–0.39) but a significant reduction in DCP post-RRD repair (MD: − 2.83%, 95% CI: − 5.04 to − 0.63). Substantial heterogeneity was noted (SCP: I^2^ = 97.5%; DCP: I^2^ = 98.6%).

### Perfusion density in choriocapillaris (CC)

**Fig. 8:**
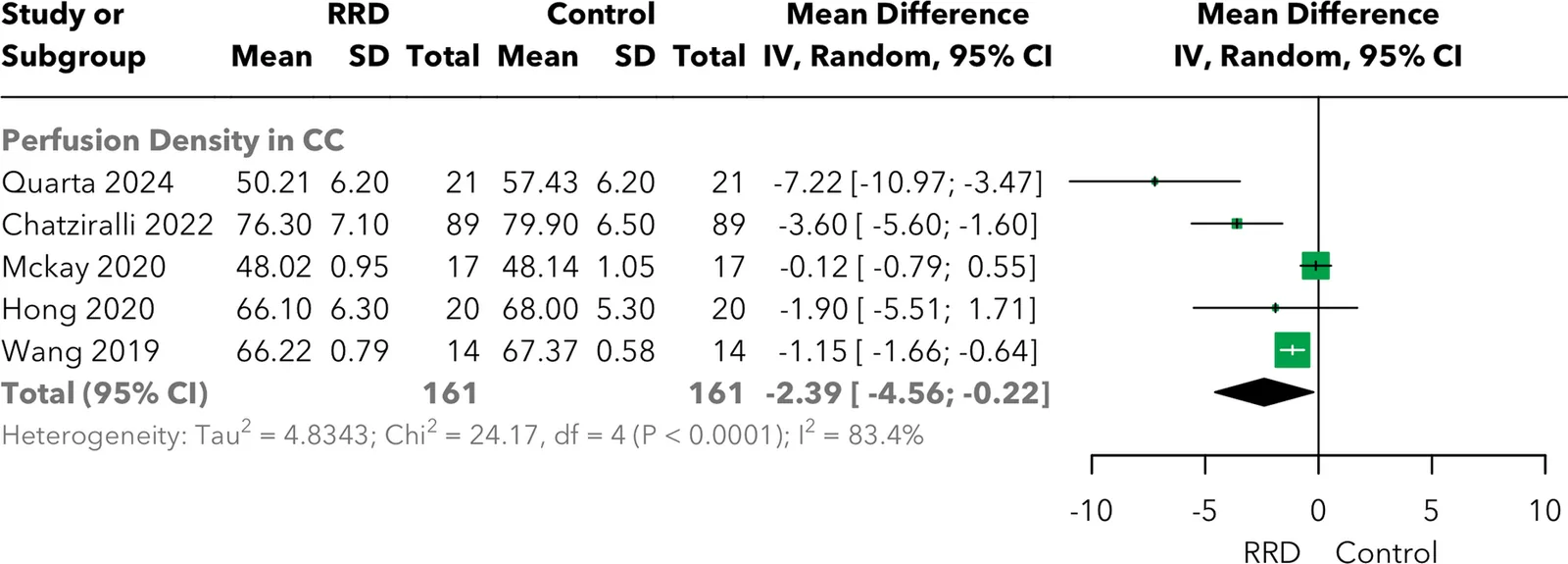
Perfusion density in choriocapillaris (CC)

CC perfusion density analysis in the macula-OFF group (n = 161 eyes) was assessed across five studies. As demonstrated in Fig. 8, there was significant post-surgical reduction (MD: − 2.39%, 95% CI: − 4.56 to − 0.22) with high heterogeneity (I^2^ = 83.4%).

### Best-corrected visual acuity (BCVA)

**Fig. 9:**
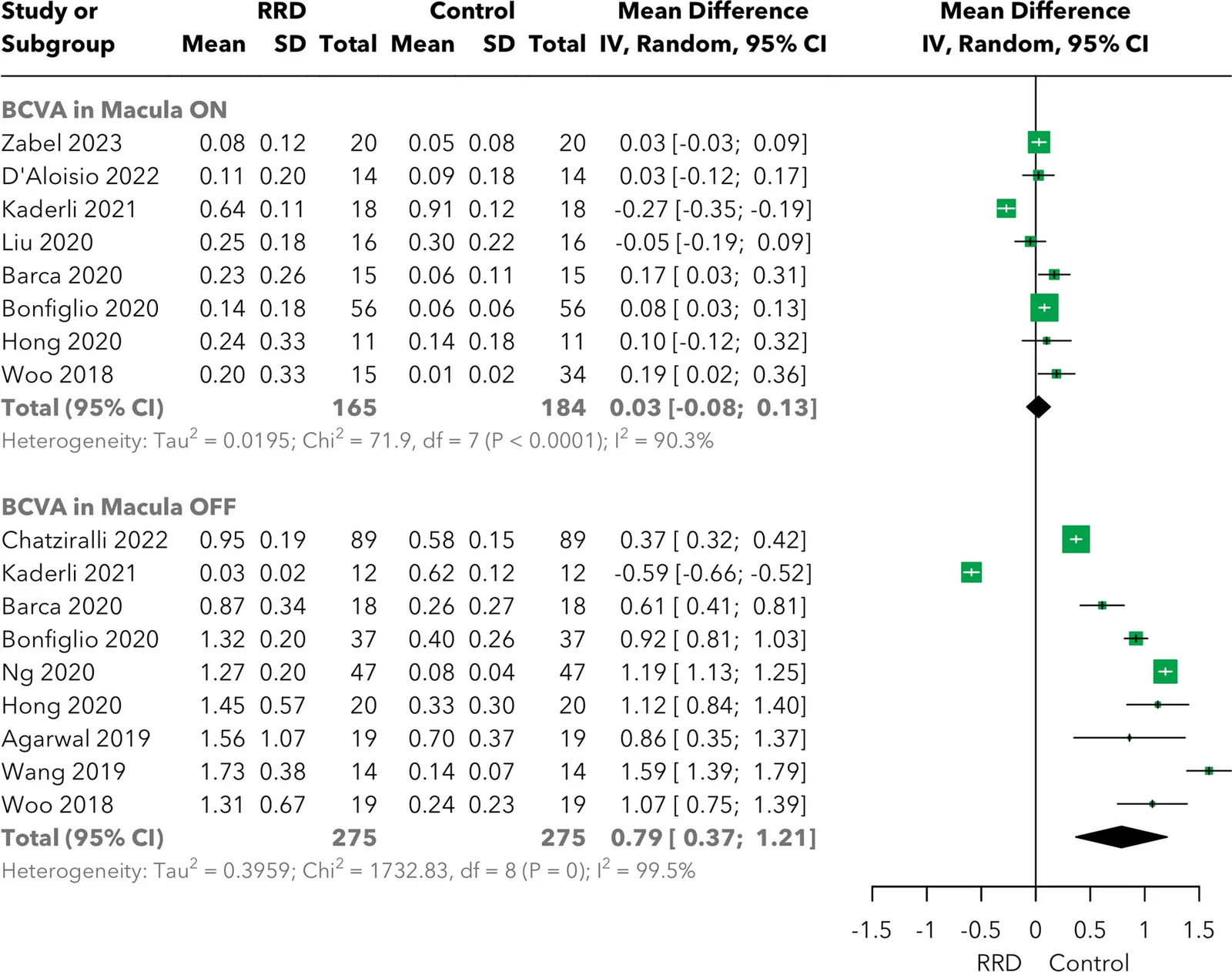
Best-corrected visual acuity (BCVA) in macula-ON and macula-OFF groups

BCVA analysis in the macula-ON group (n = 165 eyes) was evaluated, and as presented in Fig. 9, there was no significant post-surgical improvement (MD: 0.03 logMAR, 95% CI: − 0.08–0.13; I^2^ = 90.3%).

The macula-OFF group analysis (n = 275 eyes, nine studies) demonstrated significant postoperative BCVA improvement (MD: 0.79 logMAR, 95% CI: 0.37–1.21), albeit with substantial heterogeneity (I^2^ = 99.5%).

### Sensitivity analysis

A leave-one-out (L1O) sensitivity analysis was conducted by sequentially removing one study at a time and re-estimating the pooled effect for each outcome; full results are presented in Table S2. In the macula-ON group, sequential exclusion of individual studies did not materially alter the direction or statistical significance of the pooled estimates for FAZ area, FVD, PFVD, or BCVA, confirming the stability of these findings. In the macula-OFF group, outcomes characterized by high heterogeneity—including FAZ area in both plexuses, FVD, and PFVD in SCP—showed variability in effect magnitude upon individual study removal, yet the direction of the effect remained consistent throughout. The significant reduction in PFVD within the DCP and the significant improvement in BCVA in the macula-OFF group were preserved upon removal of any single study. For CC perfusion density, the pooled estimate remained statistically significant regardless of which study was excluded. These findings indicate that no single study exerts a disproportionate influence on the overall conclusions, though the substantial heterogeneity in several macula-OFF outcomes should be considered when interpreting the pooled effect magnitudes.

### Certainty of evidence (GRADE)

The certainty of evidence was assessed using the Grading of Recommendations, Assessment, Development and Evaluations (GRADE) framework for each primary outcome (Table S5). Because all included studies were observational in design, the body of evidence was initiated at low certainty. Certainty was further downgraded for inconsistency in outcomes demonstrating high heterogeneity (I^2^ > 50%), particularly in the macula-OFF group where I^2^ exceeded 80% for most parameters. Publication bias, identified via major asymmetry in the LFK index for CC perfusion density and selected vessel density outcomes, provided additional grounds for downgrading. Across all outcomes, the overall certainty of evidence was rated as *very low*, with the exception of FAZ area and FVD in the macula-ON group (rated *low*), where heterogeneity was minimal. These ratings indicate that pooled estimates should be interpreted with caution, as the true effect may differ substantially from the reported values.

### Publication bias and quality assessment

The assessment of potential publication bias demonstrated varying degrees of asymmetry across different outcome measures. Major asymmetry was detected in several parameters: perfusion density in CC (LFK= − 4.73), FVD in DCP with macula-OFF (LFK= − 4.36), PFVD in DCP with macula-ON (LFK = 2.94), PFVD in SCP with macula-OFF (LFK = 2.94), and PFVD in DCP with macula-OFF (LFK = 2.75). Minor asymmetry was observed in BCVA with macula-OFF (LFK = 1.71), FVD in SCP with macula-OFF (LFK = 1.82), FAZ area in SCP with macula-OFF (LFK= − 1.75), FAZ area in DCP with macula-OFF (LFK= − 1.54), FAZ area in DCP with macula-ON (LFK= − 1.54), and FVD in both plexuses with macula-ON (SCP: LFK=1.18; DCP: LFK = 1.35). No asymmetry was detected in FAZ area in SCP with macula-ON (LFK = 0.14), PFVD in SCP with macula-ON (LFK= − 0.48), and BCVA in macula-ON (LFK = 0.81). Egger’s regression test did not reveal significant publication bias for any of the assessed outcomes (all *p* > 0.05). These findings suggest that some caution should be exercised when interpreting the pooled estimates, particularly for parameters showing major asymmetry. DOI plots for each analyzed subgroup are provided in Table S3.

Quality assessment results (Table S4) indicate that the studies received a score ranging from 6 to 8 stars, with an average score of 7.45. This suggests that the studies were of generally good quality, with most studies showing strong methodological design and execution.

## Discussion

Our meta-analysis aimed to evaluate changes in macular perfusion parameters and visual outcomes following rhegmatogenous retinal detachment (RRD) repair. Our findings indicate an enlargement of the FAZ area and a decrease in PFVD and CC perfusion, without notable changes in FVD, alongside significant improvement in BCVA in macula-OFF group post-RRD repair.

We observed a significant difference in macular perfusion between the macula-OFF and macula-ON groups. Specifically, the FAZ area within the DCP was substantially enlarged in the macula-OFF group compared to the macula-ON cohort. Macula-OFF detachments may lead to more pronounced ischemic damage within deep foveal region. These findings align with previous studies indicating that macula-OFF RRD can result in increased FAZ areas due to prolonged ischemia and subsequent capillary dropout [5, 15]. Several studies have shown the role of the retinal DCP in nourishing the outer retina as well as the photoreceptors [20, 34]. Following prolonged retinal detachment, there is a progressive degeneration of the photoreceptors and outer retina [16]. When retinal reattachment occurs, incomplete regeneration of the outer retina may occur leading to a decrease in the trophic factors such as VEGF which maintain the DCP resulting in DCP impairment. Therefore, we suggest that the enlargement of the FAZ area in the DCP and visual outcomes is linked to the extent of degeneration, with less degeneration correlating with fewer vascular changes and better visual outcomes.

While we found no significant change in foveal VD following RRD repair, we observed a significant reduction in PFVD specifically in the DCP region. Christou et al. [24] attributed inconsistent findings regarding foveal VD changes to the fovea’s relatively avascular nature. They proposed that PFVD reduction could result from inflammatory changes triggered by Müller cell activation [35] and hypoxia-induced by inflammatory mediators in subretinal fluid [36]. In our study, the perfusion density in CC showed a significant reduction post-RRD repair. A similar process occurs in the CC as the DCP. As the outer retina degenerates, the need for perfusion decreases, leading to diminished trophic factors that sustain the CC. The CC degeneration results from a lack of these trophic factors, particularly VEGF, which are essential for maintaining choroidal vasculature [37]. This emphasizes the interdependent relationship between retinal health and choriocapillaris perfusion. Additionally, Wang et al.’s [5] investigation suggests that postoperative macular perfusion gradually improves as the retina reattaches and flattens, with variations in recovery speed between retinal and choroidal circulation. These findings highlight the interrelation between retinal and choroidal circulation during postoperative recovery and emphasize the importance of addressing choroidal vascular changes to enhance visual outcomes in retinal pathologies.

Postoperative improvements in best-corrected visual acuity (BCVA) were more substantial in macula-OFF patients, likely due to the greater potential for visual recovery following reattachment in cases where the macula was initially involved. However, the relationship between the FAZ area and BCVA is intricate. While some studies have reported an inverse correlation between FAZ size and visual acuity [34], others have found no significant association [5, 15]. Conversely, Diaz-Aljaro et al. [31] identified preoperative BCVA as the sole macular perfusion parameter predictive of favorable postoperative visual outcomes; higher preoperative BCVA was linked to a greater likelihood of achieving good visual results (odds ratio: 11.06; p = 0.0037). These conflicting findings may stem from differences in study design, imaging protocols, and patient populations.

Our study builds upon Chen et al.’s [38] work by incorporating visual acuity assessment and including updated studies from 2022 onward, enhanced by subgroup analysis. However, several limitations exist: variability in surgical timing, techniques, and OCTA examination protocols contributed to heterogeneity. Most studies had relatively small sample sizes, and variations in software, layer segmentation, and area selection during OCTA measurement added complexity. The relationship between RRD and visual outcomes involves multiple factors beyond vascular changes, including inflammatory mediators and anatomical alterations. Our methodology cannot establish direct causality between vascular changes and visual outcomes, and the observed associations should be interpreted as correlative rather than causative relationships. In addition, we were unable to perform a meta-regression analysis due to the complex nature of the subgroups and the heterogeneity in effect sizes across studies. The lack of standardized data reporting and inherent differences among subgroups made it challenging to model a relationship between dependent variables and corresponding covariates.

## Conclusion

In conclusion, this comprehensive meta-analysis demonstrates persistent alterations in macular perfusion parameters following RRD repair, particularly in macula-OFF cases. The enlarged FAZ area and reduced vascular density in both superficial and deep capillary plexuses suggest lasting microstructural changes despite successful anatomical reattachment. While significant BCVA improvement in macula-OFF cases indicates functional recovery, the persistent microvascular changes highlight the importance of early intervention. These findings underscore the value of OCTA in post-operative assessment and suggest that retinal perfusion status may serve as a prognostic indicator for visual outcomes. Future longitudinal studies with standardized OCTA protocols could further elucidate the temporal dynamics of macular perfusion recovery post-RRD repair and explore causative associations between these perfusion parameters.

## Supplementary Information

Below is the link to the electronic supplementary material.

Supplementary file1 (DOCX 544542 KB)

## Data Availability

The data that support the findings of this study are available from the corresponding author upon reasonable request.
