# Supplementary material for: Macular perfusion analysis using optical coherence tomography angiography post rhegmatogenous retinal detachment repair: a systematic review and meta-analysis
Source: Int Ophthalmol. 2026 Aug 20;46(1):336. doi: 10.1007/s10792-026-04210-8 (PMC13493483; doi:10.1007/s10792-026-04210-8)
Supplement: Supplementary file 1 [file 10792_2026_4210_MOESM1_ESM.docx]

**Supplementary Material**

**Evaluating Macular Perfusion Outcomes Post-Surgical Intervention for Rhegmatogenous Retinal Detachment Using OCTA: A Systematic Review and Meta-Analysis**

The authors have provided this supplemental material to give readers additional information about their work.

**Table S1:** Detailed Search Strategy of each database

| **Database** | **S. No.** | **Search String** | **Search Results** |
| --- | --- | --- | --- |
| **PubMed (MEDLINE)** | 1 | ("Optical Coherence Tomography Angiography"[All Fields] OR "OCTA"[All Fields] OR "angio-OCT"[All Fields] OR "OCT Angiography"[All Fields]) | 13,941 |
|  | 2 | ("Vitrectomy"[Mesh] OR "Vitrectomy"[All Fields] OR "Scleral Buckling"[Mesh] OR "Scleral Buckling"[All Fields] OR "pneumatic retinopexy") | 26,326 |
|  | 3 | ("Rhegmatogenous Retinal detachment"[All Fields] OR "Retinal detachment"[MeSH]) | 30,425 |
|  | 4 | 1-3 | 79 |
| **Scopus** | 5 | "Optical Coherence Tomography Angiography" OR "OCTA" | 63,089 |
|  | 6 | "Retinal Detachment" | 60,093 |
|  | 7 | 5-6 | 1,754 |
| **Cochrane Library** | 8 | "Optical Coherence Tomography Angiography" OR "OCTA" | 297 |
|  | 9 | "Retinal Detachment" | 1,298 |
|  | 10 | 8-9 | 239 |
| **Total** |  | **4, 7, 10** | **2,072** |

**Table S2:** Leave-one-out sensitivity (L1O) analysis

| **S2.1** FAZ ON SCP L1O |  |
| --- | --- |
| **S2.2** FAZ ON DCP L1O |  |
| **S2.3** FAZ OFF SCP L1O |  |
| **S2.4** FAZ OFF DCP L1O |  |
| **S2.5** FVD ON SCP L1O |  |
| **S2.6** FVD ON DCP L1O |  |
| **S2.7** FVD OFF SCP L1O |  |
| **S2.8** FVD OFF DCP L1O |  |
| **S2.9** PFVD ON SCP L1O |  |
| **S2.10** PFVD ON DCP L1O |  |
| **S2.11** PFVD OFF SCP L1O |  |
| **S2.12** PFVD OFF DCP L1O |  |
| **S2.13** CC Perfusion Density L1O |  |
| **S2.14** BCVA ON L1O |  |
| **S2.15** BCVA OFF L1O |  |

**Table S3:** DOI Plots

| **S3.1** FAZ ON SCP DOI Plot |  |
| --- | --- |
| **S3.2** FAZ ON DCP DOI Plot |  |
| **S3.3** FAZ OFF SCP DOI Plot |  |
| **S3.4** FAZ OFF DCP DOI Plot |  |
| **S3.5** FVD ON SCP DOI Plot |  |
| **S3.6** FVD ON DCP DOI Plot |  |
| **S3.7** FVD OFF SCP DOI Plot |  |
| **S3.8** FVD OFF DCP DOI Plot |  |
| **S3.9** PFVD ON SCP DOI Plot |  |
| **S3.10** PFVD ON DCP DOI Plot |  |
| **S3.11** PFVD OFF SCP DOI Plot |  |
| **S3.12** PFVD OFF DCP DOI Plot |  |
| **S3.13** CC Perfusion Density DOI Plot |  |
| **S3.14** BCVA ON DOI Plot |  |
| **S3.15** BCVA OFF DOI Plot |  |

**Table S4:** Quality Assessment of Included Studies

| **Study** | **Selection** |  |  |  | **Comparability** | **Outcome** |  |  | | **Total Score*** |
| --- | --- | --- | --- | --- | --- | --- | --- | --- | --- | --- |
|  | **Representativeness of the exposed cohort** | **Selection of the non-exposed cohort** | **Ascertainment of exposure** | **Demonstration that outcome of interest was not present at the start of the study** | **Comparability of cohorts based on the design or analysis** | **Assessment of outcome** | **Was follow-up long enough for outcomes to occur** | **Adequacy of follow-up of cohorts** |  | |
| Diaz-Aljaro 2024 | 1 | 1 | 1 | 1 | 1 | 1 | 1 | 1 | 8 | |
| Quarta et al. 2024 | 1 | 1 | 1 | 1 | 1 | 1 | 1 | 1 | 8 | |
| Shaheen et al. 2024 | 1 | 1 | 1 | 1 | 1 | 1 | 0 | 1 | 7 | |
| Machairoudia et al. 2023 | 1 | 1 | 1 | 1 | 1 | 1 | 1 | 1 | 8 | |
| Zabel et al. 2023 | 1 | 1 | 1 | 1 | 1 | 1 | 1 | 1 | 8 | |
| Chatziralli et al. 2022 | 1 | 1 | 1 | 1 | 1 | 1 | 1 | 0 | 7 | |
| Çetinkaya Yaprak et al. 2021 | 1 | 1 | 1 | 1 | 1 | 1 | 1 | 1 | 8 | |
| Christou et al. 2021 | 1 | 1 | 1 | 1 | 0 | 1 | 0 | 1 | 6 | |
| D'Aloisio et al. 2022 | 1 | 1 | 1 | 1 | 1 | 1 | 1 | 1 | 8 | |
| Kaderli et al. 2021 | 1 | 1 | 1 | 1 | 1 | 1 | 1 | 1 | 8 | |
| Lee and Park et al. 2021 | 1 | 1 | 1 | 1 | 1 | 1 | 1 | 1 | 8 | |
| Liu et al. 2021 | 1 | 0 | 1 | 1 | 1 | 1 | 0 | 1 | 6 | |
| Barca et al. 2020 | 1 | 0 | 1 | 1 | 1 | 1 | 1 | 1 | 7 | |
| Bonfiglio et al. 2020 | 1 | 1 | 1 | 1 | 1 | 1 | 0 | 1 | 7 | |
| Hong et al. 2020 | 1 | 1 | 1 | 1 | 1 | 1 | 1 | 1 | 8 | |
| McKay et al. 2020 | 1 | 1 | 1 | 1 | 1 | 1 | 0 | 1 | 7 | |
| Ng et al. 2020 | 1 | 1 | 1 | 1 | 1 | 1 | 1 | 1 | 8 | |
| Agarwal et al. 2019 | 1 | 1 | 1 | 1 | 1 | 1 | 1 | 0 | 7 | |
| Wang et al. 2019 | 1 | 1 | 1 | 1 | 1 | 1 | 0 | 1 | 7 | |
| Woo et al. 2018 | 1 | 1 | 1 | 1 | 1 | 1 | 1 | 1 | 8 | |
| *Maximum score is 9. Scores <6 were classified as low-quality studies, 6 to 7 as intermediate-quality studies, and 8 to 9 as high-quality studies. Reference: Wells G, Shea B, O'Connell D, Peterson J, Welch V, Losos M, et al. The Newcastle-Ottawa scale (NOS) for assessing the quality of nonrandomised studies in meta-analysis. <https://www.ohri.ca/programs/clinical_epidemiology/oxford.asp>. Accessed 2 March 2024. | | | | | | | | | | |
|  |  |  |  |  |  |  |  |  |  | |
|  |  |  |  |  |  |  |  |  |  |  |

**Table S5:** GRADE Summary of Findings

| **Outcome** | **Studies (n)** | **Participants (RRD eyes)** | **Risk of Bias** | **Inconsistency** | **Indirectness** | **Imprecision** | **Publication Bias** | **Overall Certainty** | **Pooled MD (95% CI)** | **Significant?** |
| --- | --- | --- | --- | --- | --- | --- | --- | --- | --- | --- |
| FAZ area – SCP (macula-ON) | 7 | 274 | Not serious | Not serious (I²=49%) | Not serious | Not serious | Not detected (LFK=0.14) | ⊕⊕○○ **Low** | 0.01 mm² (−0.03 to 0.04) | No |
| FAZ area – DCP (macula-ON) | 5 | 274 | Not serious | Not serious (I²=0%) | Not serious | Not serious | Minor (LFK=−1.54) | ⊕⊕○○ **Low** | 0.03 mm² (−0.01 to 0.07) | No |
| FAZ area – SCP (macula-OFF) | 10 | 441 | Not serious | Serious (I²=84%) | Not serious | Not serious | Minor (LFK=−1.75) | ⊕○○○ **Very Low** | 0.06 mm² (0.02 to 0.09) | Yes |
| FAZ area – DCP (macula-OFF) | 7 | 287 | Not serious | Serious (I²=89%) | Not serious | Not serious | Minor (LFK=−1.54) | ⊕○○○ **Very Low** | 0.12 mm² (0.04 to 0.19) | Yes |
| FVD – SCP (macula-ON) | 7 | 340 | Not serious | Not serious (I²=43%) | Not serious | Not serious | Minor (LFK=1.18) | ⊕⊕○○ **Low** | −0.14% (−1.72 to 1.44) | No |
| FVD – DCP (macula-ON) | 5 | 340 | Not serious | Not serious (I²=0%) | Not serious | Not serious | Minor (LFK=1.35) | ⊕⊕○○ **Low** | −0.26% (−1.41 to 0.88) | No |
| FVD – SCP (macula-OFF) | 11 | 608 | Not serious | Very serious (I²=95%) | Not serious | Not serious | Minor (LFK=1.82) | ⊕○○○ **Very Low** | −1.05% (−4.26 to 2.16) | No |
| FVD – DCP (macula-OFF) | 9 | 608 | Not serious | Very serious (I²=96%) | Not serious | Not serious | Serious (LFK=−4.36) | ⊕○○○ **Very Low** | −2.62% (−5.28 to 0.04) | No |
| PFVD – DCP (macula-ON) | 5 | 264 | Not serious | Serious (I²=52%) | Not serious | Not serious | Serious (LFK=2.94) | ⊕○○○ **Very Low** | −1.38% (−2.72 to −0.05) | Yes |
| PFVD – DCP (macula-OFF) | 9 | 602 | Not serious | Very serious (I²=99%) | Not serious | Not serious | Serious (LFK=2.75) | ⊕○○○ **Very Low** | −2.83% (−5.04 to −0.63) | Yes |
| CC perfusion density (macula-OFF) | 5 | 161 | Not serious | Serious (I²=83%) | Not serious | Serious (small n) | Serious (LFK=−4.73) | ⊕○○○ **Very Low** | −2.39% (−4.56 to −0.22) | Yes |
| BCVA (macula-ON) | 5 | 165 | Not serious | Very serious (I²=90%) | Not serious | Not serious | Not detected (LFK=0.81) | ⊕○○○ **Very Low** | 0.03 logMAR (−0.08 to 0.13) | No |
| BCVA (macula-OFF) | 9 | 275 | Not serious | Very serious (I²=100%) | Not serious | Not serious | Minor (LFK=1.71) | ⊕○○○ **Very Low** | 0.79 logMAR (0.37 to 1.21) | Yes |
| *FAZ = foveal avascular zone; FVD = foveal vascular density; PFVD = parafoveal vascular density; CC = choriocapillaris; BCVA = best-corrected visual acuity; SCP = superficial capillary plexus; DCP = deep capillary plexus; MD = mean difference; CI = confidence interval. All studies were observational (non-randomized), initiating at Low certainty per GRADE. Certainty was downgraded for serious inconsistency (I² >50%) and/or serious publication bias (LFK index >2 or <−2). ⊕○○ Low; ⊕○○○ Very Low.* | | | | | | | | | | |
